# Vascularized Flaps as Living Bioreactors in Bone Tissue Engineering: From Biological Principles to Translational Strategies—A Narrative Review

**DOI:** 10.3390/jfb17060270

**Published:** 2026-06-01

**Authors:** Fabiana Battaglia, Michele Rosario Colonna, Emanuele Cigna, Michele Maruccia, Gabriele Delia

**Affiliations:** 1 Department of Plastic and Reconstructive Surgery, University Hospital of Messina “AOU Gaetano Martino”, 98125 Messina, Italy; 2 Plastic Surgery and Microsurgery Unit, Department of Translational Research and New Technologies in Medicine and Surgery, University of Pisa, 56122 Pisa, Italy; 3 Section of Plastic and Reconstructive Surgery, Department of Emergency and Organ Transplantation, University of Bari, 70121 Bari, Italy

**Keywords:** bone tissue engineering, vascularized flaps, arteriovenous loop, axial vascularization, scaffold-guided bone regeneration

## Abstract

**Background:** Large segmental bone defects remain a major challenge in reconstructive surgery, particularly in the presence of impaired vascularization. Despite advances in scaffold design and biomaterials, insufficient vascular supply continues to represent the primary limitation in bone tissue engineering, often leading to impaired osteogenesis and graft failure. **Objective:** This review aims to analyze the role of vascularized flaps as “living bioreactors” in bone tissue engineering, focusing on their capacity to enhance scaffold vascularization, support osteogenesis, and facilitate clinical translation. **Methods:** A narrative review was conducted through a structured search of PubMed, Scopus, and Web of Science using combinations of the following keywords: “bone tissue engineering”, “vascularized flaps”, “arteriovenous loop”, and “in vivo bioreactor”. Relevant preclinical and clinical studies were selected based on their contribution to vascularization strategies in scaffold-based bone regeneration, with the aim of illustrating the evolution and integration of these approaches. **Results:** Vascularized flaps provide an established vascular network and a biologically active microenvironment that promote scaffold integration and tissue regeneration. Periosteal flaps demonstrate strong osteogenic potential, whereas muscle and omental flaps primarily act as vascular carriers and adaptable regenerative environments. AV loop-based strategies enable intrinsic axial vascularization, ensuring rapid and homogeneous perfusion of large constructs. Hybrid approaches, including regenerative matching axial vascularization (RMAV), integrate vascularized tissues with advanced biomaterials and show promising translational outcomes. **Conclusions:** Vascularization-driven strategies represent a paradigm shift in bone tissue engineering, moving from passive scaffold implantation to actively engineered, vascularized constructs. The integration of microsurgical techniques with advanced biomaterials offers significant potential for the development of personalized and clinically applicable bone regeneration strategies.

## 1. Introduction

Large segmental bone defects remain one of the most challenging problems in reconstructive surgery, particularly when associated with compromised vascularization and impaired regenerative potential [1,2]. Current gold-standard approaches, including vascularized bone grafts and autologous tissue transfer, provide reliable outcomes but are limited by donor-site morbidity, limited tissue availability, and surgical complexity [3,4]. These challenging defects commonly arise following severe trauma, oncologic resection, chronic infection, or congenital skeletal abnormalities, often resulting in compromised vascularity and limited regenerative capacity. These limitations have driven increasing interest in bone tissue engineering strategies aimed at regenerating functional bone tissue through the integration of scaffolds, cells, and bioactive signals.

However, despite significant advances in scaffold design and biomaterials, the lack of rapid and sufficient vascularization remains the primary bottleneck in the successful translation of engineered constructs [5,6,7,8]. Cell survival and osteogenesis are critically dependent on proximity to a functional capillary network, with diffusion limits restricting viability in poorly vascularized scaffolds [9].

This limitation has driven the development of strategies aimed at integrating vascularization into scaffold-based constructs from the earliest stages of tissue formation.

Among these, the concept of the in vivo bioreactor has emerged as a paradigm-shifting approach. Rather than attempting to engineer vascularized tissue ex vivo, this strategy leverages the intrinsic regenerative capacity of the host by using vascularized tissues, particularly flaps, as biological environments for scaffold maturation [10]. In this context, vascularized flaps act not merely as carriers but as “living bioreactors”, providing a dynamic microenvironment rich in progenitor cells, growth factors, extracellular matrix components, and an established vascular network [11].

Early experimental evidence demonstrated the feasibility of this approach using periosteal flaps. In a rabbit model, Huang et al. showed that pedicled periosteal flaps combined with decellularized bone matrix scaffolds significantly enhanced bone formation, vascular density, and biomechanical strength compared to non-vascularized controls [12].

Similarly, the critical role of vascular supply has been confirmed in axial vascularization models. In a rat femoral defect model, Nau et al. demonstrated that vascularized periosteal flaps combined with β-TCP scaffolds and progenitor cells significantly improved bone mineral density, angiogenesis, and mechanical stability, while interruption of the vascular pedicle markedly impaired regeneration [13]. These findings underscore that vascularization is not merely supportive but essential for effective scaffold-based bone regeneration [14,15].

The clinical translation of the in vivo bioreactor concept was first demonstrated in a landmark study by Warnke et al., who successfully engineered and transplanted a patient-specific vascularized mandibular graft generated within a latissimus dorsi muscle flap [16]. This pioneering work established the feasibility of generating functional vascularized bone constructs in vivo, avoiding the need for traditional bone harvesting [17].

Subsequent developments have expanded this concept through the integration of arteriovenous (AV) loop-based vascularization, enabling the creation of intrinsically vascularized constructs independent of local tissue beds. The AV loop model, originally described by Erol and Spira, laid the foundation for axial vascularization strategies, later refined by Arkudas, Kneser, and colleagues to support the generation of large, transplantable tissue constructs [9,18,19].

More recently, clinically relevant large-animal models and translational studies have demonstrated the effectiveness of combining vascularized flaps with advanced scaffold technologies. Paré et al. showed successful regeneration of critical-size mandibular defects using a custom 3D-printed calcium phosphate scaffold vascularized via an arteriovenous loop, achieving complete osseointegration and lamellar bone formation at 12 months [20,21,22,23].

Similarly, scaffold-guided bone regeneration approaches incorporating corticoperiosteal flaps have demonstrated outcomes comparable to autologous grafts, highlighting the importance of sustained vascular supply in large defect healing [24].

In parallel, innovative surgical strategies such as omentum-based bioreactors and minimally invasive scaffold implantation techniques have further expanded the applicability of this concept, demonstrating successful bone formation within vascularized environments in large animal models [25,26,27].

Despite these advances, the field remains fragmented, with studies spanning diverse models, vascularization strategies, and scaffold technologies. To date, no comprehensive synthesis has specifically focused on the role of vascularized flaps as active biological systems enabling scaffold vascularization and bone regeneration.

Therefore, this review aims to analyze the role of vascularized flaps as “living bioreactors” in bone tissue engineering, focusing on their biological rationale, experimental evidence, axial vascularization strategies, and translational potential. Although several foundational studies in this field were published during the early development of vascularized bone tissue engineering, these pioneering investigations continue to represent the biological and microsurgical basis of current translational strategies. Given the interdisciplinary nature of bone tissue engineering, this review also aims to provide an integrated clinical and biological overview accessible to researchers working in biomaterials science, regenerative medicine, tissue engineering, and reconstructive surgery.

## 2. Materials and Methods

A comprehensive literature search was conducted using PubMed, Scopus, and Web of Science databases. The objective of the search strategy was to identify preclinical, translational, and clinical studies investigating the role of vascularized flaps, arteriovenous loops, axial vascularization, and in vivo bioreactor models in bone tissue engineering. Given the narrative nature of this review, no formal systematic review protocol or PRISMA methodology was applied.

The search terms included combinations of Medical Subject Headings (MeSH) and free-text terms, such as “bone tissue engineering”, “vascularized flaps”, “vascularized periosteal flap”, “periosteal flap”, “muscle flap”, “omental flap”, “arteriovenous loop”, “axial vascularization”, “intrinsic vascularization”, “in vivo bioreactor”, “scaffold-guided bone regeneration”, “3D-printed scaffold”, and “regenerative matching axial vascularization”. These terms were used both independently and in combination with one another.

The selection procedure included evaluation of the title, abstract, and full text of potentially relevant articles to assess their suitability and contribution to the objectives of this review. Studies were considered eligible if they addressed vascularization strategies in scaffold-based bone regeneration, described the use of vascularized tissues as biological environments for bone formation, or provided relevant experimental or clinical evidence on flap-assisted or AV loop-based bone tissue engineering.

Preclinical studies, large-animal models, translational studies, clinical case reports, feasibility studies, and relevant reviews were included when they provided significant insights into the biological mechanisms, technical principles, or clinical applicability of vascularized constructs. Duplicates, conference abstracts, book chapters, and articles not directly related to vascularization-driven bone regeneration were excluded. Specifically, studies focused exclusively on soft-tissue reconstruction without bone regeneration endpoints or without scaffold-based regenerative applications were not considered eligible. Following implantation within vascularized biological environments, engineered constructs are typically evaluated in preclinical small- and large-animal models through assessment of angiogenesis, scaffold integration, bone formation, biomechanical stability, and translational feasibility.

The included literature was analyzed and organized thematically, focusing on biological principles, vascularized flap types, axial vascularization strategies, hybrid scaffold-based approaches, and translational implications.

## 3. Discussion

The evidence presented in the literature highlights vascularization as the critical determinant of success in bone tissue engineering [28,29]. Therefore, a mechanistic understanding of how vascularized strategies support scaffold integration and osteogenesis is essential. In this context, vascularized flaps emerge as key biological systems capable of actively driving tissue regeneration.

### 3.1. Biological Basis of Vascularized Flaps as Living Bioreactors

A major limitation in bone tissue engineering is the inability to achieve rapid and sufficient vascularization within scaffold-based constructs. It is well established that oxygen and nutrient diffusion is limited to a distance of approximately 200–500 μm from the nearest capillary, beyond which cell survival is compromised [7,30,31,32]. Consequently, large scaffolds frequently develop central necrosis, impaired osteogenesis, and ultimately fail to integrate with host tissue [33,34].

Traditional strategies rely on extrinsic vascularization, whereby neovessels infiltrate the scaffold from surrounding tissues. However, this process is inherently slow and often insufficient for large constructs, leading to delayed perfusion and incomplete regeneration. In contrast, intrinsic vascularization, achieved through the introduction of a defined vascular axis, allows rapid and homogeneous perfusion throughout the construct, significantly enhancing tissue viability and regenerative outcomes [9,18,19,35].

Vascularized flaps offer a unique biological solution to this challenge. Unlike passive scaffold implantation, they provide an already established vascular network capable of immediate perfusion, thereby overcoming the temporal limitations associated with angiogenic ingrowth. In addition to vascular supply, flaps contribute a complex biological microenvironment composed of progenitor cells, extracellular matrix components, and a wide range of growth factors that collectively support osteogenesis [14,15,27].

In most bone tissue engineering strategies, biomaterial scaffolds are implanted within, wrapped by, or directly associated with vascularized flaps, allowing immediate interaction between the scaffold and the vascularized biological environment. This configuration promotes angiogenic ingrowth, nutrient diffusion, progenitor cell recruitment, and progressive osteogenic maturation of the construct.

Among the different flap types, the periosteum represents one of the most potent biological interfaces. Its intrinsic osteogenic capacity, combined with a dense vascular network, makes it an ideal candidate for in vivo bioreactor strategies [36,37]. Huang et al. demonstrated that pedicled periosteal flaps combined with decellularized bone matrix significantly enhanced bone formation, vascular density, and biomechanical strength compared to non-vascularized controls [12,36]. Similarly, Nau et al. showed that the addition of mesenchymal stem cells and endothelial progenitor cells to β-TCP scaffolds in combination with a vascularized periosteal flap resulted in significantly improved bone mineral density, angiogenesis, and biomechanical stability, while interruption of the vascular pedicle markedly impaired these outcomes [13,28].

Beyond periosteal flaps, other vascularized tissues such as muscle and omentum have also been successfully employed as biological incubators for scaffold maturation. These tissues provide a highly vascularized environment capable of supporting ectopic bone formation and have been used in both experimental and translational settings. For instance, omentum-based bioreactor models have demonstrated the feasibility of scaffold implantation followed by delayed flap transfer, enabling minimally invasive approaches to generate vascularized bone constructs [25].

A key conceptual advance in this field is the recognition that vascularized flaps function not simply as structural carriers but as dynamic biological systems. They actively regulate tissue regeneration through continuous interactions between vascular supply, cellular components, and biochemical signaling pathways. This concept is further supported by evidence showing that interruption of vascular flow dramatically reduces bone formation, even in the presence of osteoconductive scaffolds and osteogenic cells [13].

The development of arteriovenous (AV) loop models has further clarified the mechanisms underlying intrinsic vascularization. By establishing a central vascular axis within an isolated chamber [38], AV loop systems, based on the microsurgical creation of an arteriovenous shunt acting as a central vascular axis, enable the generation of fully vascularized tissue constructs independent of the surrounding tissue bed. Early foundational work by Kneser et al. demonstrated the feasibility of inducing axial vascularization within an in vivo bioreactor [18], while subsequent studies by Arkudas et al. showed that this approach allows the generation and transplantation of large tissue constructs with homogeneous vascularization and improved tissue survival [9,19,39].

More recently, the integration of vascularized flaps with advanced scaffold technologies has further reinforced this paradigm. Large-animal and translational studies have demonstrated that combining intrinsic vascularization with scaffold-guided regeneration significantly improves outcomes in critical-size defects [20,24].

Collectively, these observations support a paradigm shift in bone tissue engineering: from scaffold-centered approaches to vascularization-driven strategies, in which the presence of a functional vascular network is considered the primary determinant of success. Within this framework, vascularized flaps emerge as ideal platforms for tissue regeneration, acting as living bioreactors.

### 3.2. Biological Flaps as In Vivo Bioreactors: Periosteum, Muscle and Omentum

In reconstructive surgery, a vascularized flap refers to a segment of tissue transferred together with its intrinsic blood supply to restore tissue defects. Depending on their composition, flaps may include periosteum, muscle, fascia, adipose tissue, or composite tissues, each providing distinct biological and vascular properties relevant to tissue engineering applications.

Vascularized flaps represent heterogeneous biological systems, each characterized by distinct cellular composition, vascular architecture, and regenerative potential [40]. In the context of bone tissue engineering, these differences are highly relevant, as they directly influence the ability of the flap to support scaffold vascularization and osteogenesis. Among the various options, periosteal, muscle, and omental flaps have emerged as the most extensively investigated biological platforms for in vivo bioreactor strategies.

**Figure 1:**
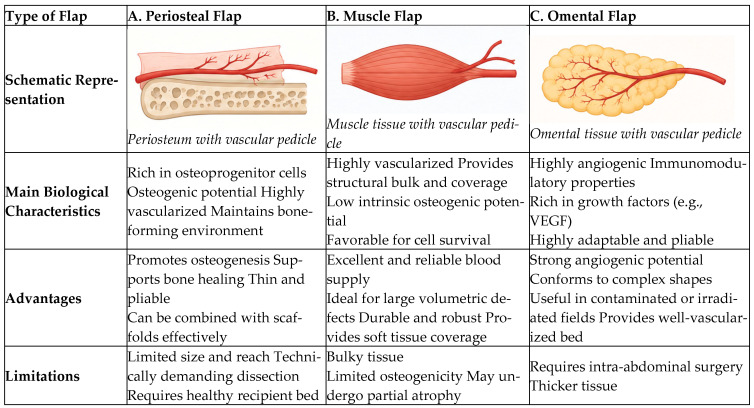
Main types of vascularized flaps used as in vivo bioreactors in bone tissue engineering.

The main biological characteristics, advantages, and limitations of the principal vascularized flaps used as in vivo bioreactors in bone tissue engineering are summarized in Figure 1.

### 3.3. Periosteal Flaps: The Osteogenic Gold Standard

The periosteum is widely recognized as the most effective biological interface for bone regeneration due to its intrinsic osteogenic capacity and dense vascular network [41]. It contains a rich population of mesenchymal progenitor cells capable of differentiating into osteoblasts, as well as an abundant microvascular system that supports rapid tissue perfusion [42].

Experimental evidence strongly supports the superiority of periosteal flaps in scaffold-based bone regeneration. Huang et al. demonstrated that pedicled periosteal flaps combined with decellularized bone matrix significantly enhanced bone formation, vascular density, and mechanical strength compared to non-vascularized controls [12]. Similarly, Nau et al. showed that the integration of β-TCP scaffolds with mesenchymal stem cells and endothelial progenitor cells within a vascularized periosteal flap resulted in significantly improved bone mineral density, angiogenesis, and biomechanical stability [13].

These findings are further supported by large-animal and translational models. Sparks et al. demonstrated that corticoperiosteal flaps combined with 3D-printed mPCL-TCP scaffolds enabled regeneration of critical-size bone defects with outcomes comparable to autologous grafts, both in preclinical and clinical settings [24]. Likewise, Paré et al. reported successful regeneration of mandibular defects using axially vascularized calcium phosphate scaffolds integrated with periosteal vascularization, achieving complete osseointegration and lamellar bone formation [20].

Collectively, these studies establish periosteal flaps as the gold standard biological platform for in vivo bioreactor-based bone tissue engineering.

### 3.4. Muscle Flaps: Vascular Carriers and Inductive Environments

Muscle flaps, although lacking intrinsic osteogenic capacity, provide a highly vascularized and metabolically active environment that supports tissue regeneration [43,44]. Their role in bone engineering is primarily that of a vascular carrier, capable of delivering blood supply and facilitating the recruitment of circulating progenitor cells.

The clinical relevance of muscle-based bioreactors was first demonstrated in the landmark study by Warnke et al., who successfully engineered a vascularized mandibular graft within a latissimus dorsi muscle flap, combining scaffold, growth factors, and bone marrow-derived cells [16]. This study provided the first proof-of-concept that complex, patient-specific bone constructs could be generated in vivo and subsequently transplanted.

Further experimental and translational studies have demonstrated that muscle tissue can support ectopic bone formation when combined with appropriate osteoinductive stimuli. In vivo bioreactor approaches have shown that vascularized muscle environments can sustain the maturation of scaffold-based constructs and promote de novo bone formation, particularly when combined with osteogenic cells and bioactive factors [18].

Thus, muscle flaps can be considered biological amplifiers, in which vascularization is readily available, but osteogenesis depends on the addition of exogenous biological signals.

### 3.5. Omental Flaps: Highly Vascularized and Adaptable Bioreactors

The omentum represents a unique biological platform characterized by exceptional vascularity, immunomodulatory properties, and high adaptability. Its rich angiogenic potential and capacity to conform to complex geometries make it particularly suitable for scaffold-based tissue engineering [45,46].

Recent studies have demonstrated the feasibility of using the omentum as an ectopic bioreactor for bone regeneration. Naujokat et al. described a minimally invasive approach involving robot-assisted scaffold implantation within the omentum, followed by delayed flap harvesting, demonstrating successful scaffold integration and bone formation [25]. Similarly, Naujokat et al. and related experimental models have shown that omental flaps can support the generation of vascularized bone constructs while reducing surgical morbidity.

In addition, Watson et al. demonstrated that periosteum-driven bone formation within a vascularized environment adjacent to rib periosteum could be modulated by local biological conditions, including infection, further highlighting the dynamic nature of these systems [47].

Compared to periosteal flaps, omental flaps exhibit lower intrinsic osteogenic potential but compensate through their exceptional vascular supply and biological plasticity, making them particularly useful in complex or minimally invasive reconstructive strategies.

### 3.6. Comparative Perspective and Biological Hierarchy

Taken together, these findings suggest a functional hierarchy among biological flaps used as in vivo bioreactors. [48] Table 1.

This hierarchy highlights that the success of scaffold-based bone regeneration depends not only on vascularization but also on the biological identity of the vascularized tissue used to support the construct.

The main preclinical and clinical studies investigating vascularized flaps and axial vascularization strategies in bone tissue engineering are summarized in Table 2.

### 3.7. Arteriovenous Loop and Axial Vascularization Strategies

Arteriovenous (AV) loop-based vascularization represents one of the most advanced strategies for achieving controlled intrinsic vascularization in bone tissue engineering. Unlike traditional flap-based approaches, which rely on pre-existing vascularized tissues, AV loop systems enable the generation of a de novo vascular axis within a defined three-dimensional environment, thereby allowing precise and homogeneous vascularization of scaffold-based constructs.

The concept of axial vascularization using an AV loop was first introduced by Erol and Spira, who demonstrated that the creation of an arteriovenous fistula could induce vascular sprouting and the formation of a new capillary network within an isolated chamber [56,57]. This foundational work established the principle that vascularization can be actively engineered rather than passively awaited.

**Figure 2:**
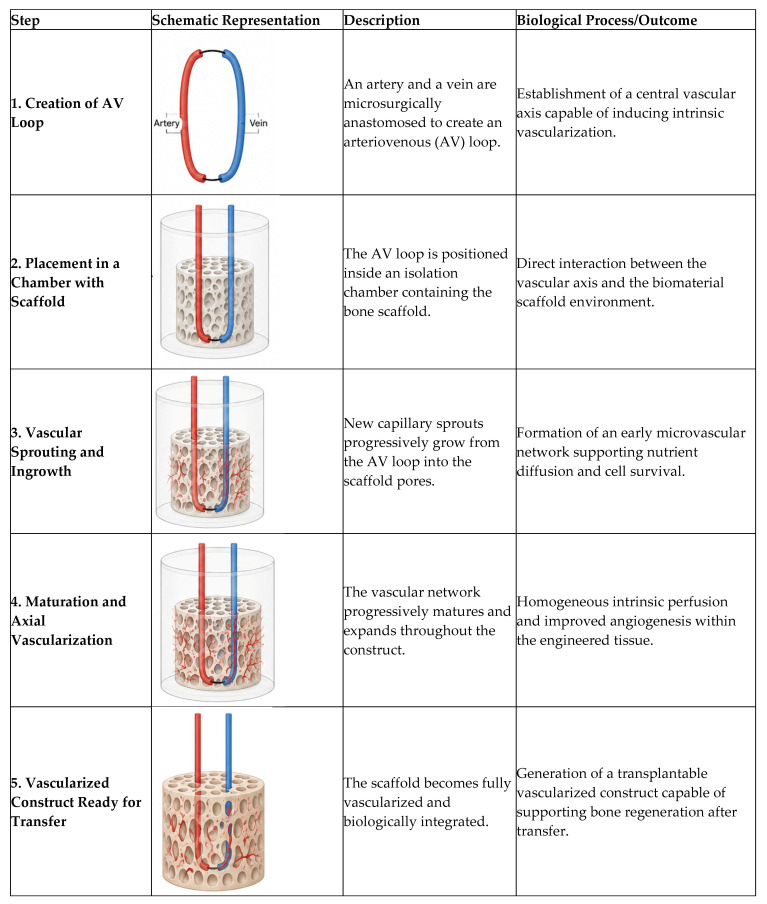
Sequential stages of AV loop–based axial vascularization in bone tissue engineering, from microsurgical AV loop creation to the generation of a fully vascularized scaffold suitable for transplantation and bone regeneration.

Subsequent experimental studies refined this concept through the development of the isolation chamber model, enabling the generation of vascularized tissue constructs independent of surrounding tissues. A schematic overview of the AV loop–based intrinsic vascularization strategy is presented in Figure 2.

Kneser et al. demonstrated that AV loop-based systems can successfully induce axial vascularization within biomaterial scaffolds, leading to the formation of transplantable tissue constructs [18]. Similarly, Arkudas et al. showed that combining AV loop vascularization with scaffold-based tissue engineering significantly enhances both angiogenesis and bone formation, particularly when integrated with mesenchymal stromal cells [9,19].

A key advantage of AV loop systems lies in their ability to provide intrinsic vascularization, establishing a central vascular axis that ensures rapid and uniform perfusion throughout the construct. This approach effectively overcomes diffusion-related limitations that typically impair large scaffold constructs and represents a critical factor in preventing central necrosis and graft failure [9,18,19].

The translational potential of AV loop-based strategies has been increasingly demonstrated in large-animal models. Paré et al. reported successful regeneration of critical-size mandibular defects using a custom 3D-printed calcium phosphate scaffold vascularized via an AV loop, achieving complete osseointegration and the formation of mature lamellar bone at 12 months [20]. These findings align with the concept of regenerative matching axial vascularization (RMAV), which aims to synchronize scaffold architecture with vascular supply to optimize regenerative outcomes.

Further advancements have been achieved by integrating AV loop systems with in vivo bioreactor strategies and advanced biomaterials. Falkner et al. demonstrated that AV loop-based vascularization within an isolation chamber can generate fully vascularized, transplantable soft tissue flaps in a large-animal model, characterized by homogeneous microvascular networks and successful microsurgical transfer. More recently, Mayer et al. further expanded this concept by engineering and transplanting axially vascularized and epithelialized flaps in a rat model, highlighting the translational potential of AV loop-based constructs for functional tissue reconstruction. These studies provide important mechanistic insight into the capacity of AV loop systems to generate stable and functional intrinsic vascular networks, which are critical for the success of large-scale tissue-engineered constructs [58]. Although not directly focused on bone regeneration, this model provides important mechanistic insight into the capacity of AV loop systems to generate stable and functional intrinsic vascular networks, which are critical for the success of large-scale tissue-engineered constructs [59]. This approach highlights the potential of AV loop strategies as an alternative to conventional free flaps while minimizing donor-site morbidity.

In parallel, hybrid models combining vascular pedicles and scaffold-based regeneration have further expanded the applicability of these strategies. Morrison et al. demonstrated that vascularized chamber systems can support the growth of complex tissue constructs, reinforcing the concept of controlled in vivo tissue engineering [60,61,62]. Similarly, Gonzalez Matheus et al. proposed a clinically oriented RMAV approach, integrating 3D-printed scaffolds with vascularized periosteum to enhance both vascularization and osteogenesis in large calvarial defects [55].

Collectively, these studies demonstrate that AV loop-based strategies provide a powerful platform for achieving controlled intrinsic vascularization. When integrated with biologically active flaps, these approaches enable the generation of complex, functional constructs and represent a critical step toward the clinical translation of large-scale bone tissue engineering.

### 3.8. Hybrid and Translational Strategies

Recent advances in bone tissue engineering have increasingly shifted toward hybrid strategies that integrate vascularized flaps, axial vascularization techniques, and scaffold-based regeneration. Rather than relying on a single approach, these models aim to combine the biological advantages of vascularized tissues with the structural and functional versatility of engineered scaffolds, thereby overcoming the intrinsic limitations of each individual component.

A key development in this context is the concept of regenerative matching axial vascularization (RMAV), which emphasizes the alignment between scaffold architecture and vascular supply. Sparks et al. demonstrated that the combination of corticoperiosteal flaps with 3D-printed mPCL-TCP scaffolds enables robust bone regeneration in large defects, achieving outcomes comparable to autologous grafts in both preclinical and clinical settings [24]. This approach highlights the importance of synchronizing mechanical support, osteogenic potential, and vascularization within a unified regenerative strategy.

Similarly, Gonzalez Matheus et al. translated this concept into clinical practice by combining medical-grade polycaprolactone–tricalcium phosphate scaffolds with vascularized periosteal tissue for the reconstruction of complex calvarial defects. Their results demonstrated effective integration, stable vascularization, and favorable clinical outcomes, supporting the feasibility of scaffold-guided, flap-assisted bone regeneration in humans [55].

Hybrid strategies also include the integration of biological stimulation within vascularized environments. Several studies have demonstrated that the combination of osteoconductive scaffolds such as β-TCP with osteoinductive stimuli, including BMP-2 and mesenchymal stem cells, can promote de novo bone formation when implanted in a well-vascularized environment [63,64]. In particular, vascularized muscle-based in vivo bioreactor models have highlighted the synergistic role of vascularization and osteoinductive signaling in supporting bone regeneration [16]. These findings suggest that vascularized flaps can act as amplifiers of biological stimuli, enhancing the regenerative capacity of engineered constructs.

In parallel, chamber-based models have provided further insights into controlled in vivo tissue engineering. Morrison et al. demonstrated that vascularized chamber systems can support the growth of complex tissue constructs, offering a controllable environment for studying the interaction between scaffold materials, vascularization, and cellular components [60]. These models bridge the gap between experimental vascular induction and clinically applicable reconstruction strategies.

Collectively, these hybrid approaches demonstrate that the future of bone tissue engineering lies in the integration of structural, biological, and vascular components. Rather than treating scaffolds, cells, and vascularization as separate elements, successful strategies increasingly rely on their coordinated interaction within a biologically active environment.

From a translational perspective, these developments mark a shift toward personalized and patient-specific reconstruction, in which scaffold design, vascularization strategy, and biological augmentation are tailored to the characteristics of the defect and the patient. The use of 3D printing technologies, combined with vascularized tissue transfer, allows precise anatomical reconstruction while maintaining functional vascular supply. However, despite these advances, the fabrication of integrated vascular channels at clinically viable resolution remains a major challenge in bioprinting and continues to represent a critical bottleneck for the translation of large-scale engineered constructs.

Despite these promising advances, several challenges remain, including the complexity of surgical procedures, the need for standardized protocols, and the limited availability of large-scale clinical data. Nevertheless, the convergence of vascularized flaps, AV loop systems, and advanced biomaterials represents a critical step toward the routine clinical application of tissue-engineered bone constructs.

## 4. Clinical Implications and Decision-Making

Despite the growing body of experimental and translational evidence supporting vascularization-driven bone regeneration, the clinical integration of these strategies remains limited by the lack of standardized decision-making frameworks. Current reconstructive approaches are still largely based on traditional algorithms that do not fully incorporate advances in scaffold-based tissue engineering and vascularization strategies.

The evidence reviewed in this work suggests that vascularization should be considered a primary determinant in selecting the optimal reconstructive strategy, rather than a secondary adjunct to structural reconstruction. In this context, vascularized flaps can be strategically employed not only for defect coverage but also as active biological platforms to enhance scaffold integration and osteogenesis.

From a clinical perspective, the choice of reconstructive strategy should be guided by a combination of defect characteristics, local vascular status, biological environment, and reconstructive goals. In this context, omental flaps may be particularly suitable for complex or irregular defects because of their high vascularity, adaptability, and immunomodulatory properties, although their osteogenic contribution remains indirect.

In situations where local vascularized tissues are insufficient or unavailable, axial vascularization strategies, such as arteriovenous (AV) loop-based approaches, provide a powerful alternative. By establishing an intrinsic vascular axis within the construct, these techniques enable rapid and homogeneous perfusion, overcoming the limitations of diffusion-dependent vascularization. AV loop systems are particularly relevant for large-scale or prefabricated constructs, where controlled vascularization is critical.

The most promising clinical strategies are increasingly based on hybrid approaches, combining vascularized flaps, axial vascularization techniques, and advanced scaffold technologies. These integrated models allow the simultaneous optimization of mechanical stability, biological activity, and vascular supply, representing a significant evolution from traditional reconstructive paradigms.

Based on the available evidence, a conceptual and simplified decision-making framework can be proposed to guide the selection of vascularization strategies in bone tissue engineering. To the best of our knowledge, no standardized clinical decision-making framework specifically integrating vascularized flaps, scaffold-based regeneration, and axial vascularization strategies currently exists in the literature. Therefore, the following framework should be interpreted as the authors’ conceptual synthesis of the available evidence rather than as a validated clinical protocol:

‑ Small defects with preserved vascularity → autologous bone grafting remains the standard approach
‑ Large defects with adequate soft tissue coverage → scaffold-based reconstruction with or without biological augmentation
‑ Large defects with poor vascularity or soft tissue loss → vascularized flap (preferably periosteal) combined with scaffold-based reconstruction
‑ Complex or critical-size defects → hybrid strategies integrating vascularized flaps, scaffolds, and potentially AV loop-based vascularization
‑ Extensive defects or prefabricated constructs → AV loop-based axial vascularization to ensure intrinsic and homogeneous perfusion.

A proposed decision-making framework for selecting vascularization strategies in scaffold-based bone reconstruction is summarized in Table 3.

Vascularization should not be considered an adjunct to bone tissue engineering, but rather its central driving force. The integration of vascularized biological systems with scaffold-based constructs represents the key to achieving reliable and clinically translatable bone regeneration.

## 5. Future Perspectives

Recent advances in vascularization-driven bone tissue engineering are progressively shifting the field toward personalized and clinically translatable regenerative strategies. Emerging technologies such as three-dimensional bioprinting and patient-specific scaffold fabrication may allow the development of constructs precisely tailored to the anatomical and biomechanical requirements of individual defects [65]. In this context, the integration of vascularized flaps with customized biomaterials represents a promising avenue for achieving both structural and biological optimization.

Smart biomaterials capable of modulating cellular behavior, releasing growth factors in a controlled manner, or dynamically responding to the local microenvironment may further enhance osteogenesis and vascular maturation [66,67]. In parallel, the incorporation of bioactive coatings, angiogenic molecules, and stem cell–based therapies could improve the regenerative performance of scaffold-based constructs.

Artificial intelligence (AI)-assisted scaffold design and computational modeling may also play an increasingly important role in optimizing pore architecture, vascular distribution, and mechanical properties, facilitating the development of highly efficient regenerative platforms [68]. These technologies could support the creation of predictive and patient-specific reconstruction strategies.

Another promising direction involves the refinement of prefabricated vascularized constructs generated through in vivo bioreactor systems and AV loop-based approaches. The possibility of producing transplantable, intrinsically vascularized tissues with reduced donor-site morbidity may significantly expand the clinical applicability of tissue-engineered bone reconstruction.

Despite these advances, several challenges remain before widespread clinical implementation can be achieved. Future research should focus on the standardization of surgical protocols, scaffold manufacturing processes, and outcome assessment methods, as well as on the generation of robust clinical evidence through multicenter translational studies. The convergence of microsurgery, biomaterials science, and regenerative medicine is expected to play a central role in the future evolution of reconstructive surgery.

## 6. Conclusions

In conclusion, vascularization represents the central determinant of success in bone tissue engineering, particularly in the context of large and complex defects. The evidence reviewed in this work highlights how vascularized flaps function as dynamic biological systems capable of supporting scaffold integration, enhancing osteogenesis, and overcoming the intrinsic limitations of diffusion-dependent tissue regeneration.

The integration of vascularized flaps with axial vascularization strategies, such as arteriovenous loop models, and advanced scaffold technologies has enabled the development of hybrid approaches that combine structural support, biological activity, and controlled perfusion. These strategies represent a significant step forward compared to traditional scaffold-based methods, shifting the paradigm from passive tissue regeneration to actively engineered vascularized constructs.

Importantly, the concept of vascularized flaps as “living bioreactors” provides a unifying framework for understanding the interaction between vascular supply, cellular components, and biomaterials. Within this framework, the success of regenerative strategies depends not only on scaffold design but also on the selection and optimization of the vascularized biological environment.

Future developments in this field will likely focus on the refinement of patient-specific approaches, integrating 3D printing technologies, biologically active scaffolds, and tailored vascularization strategies. The convergence of microsurgical techniques and regenerative medicine holds the potential to redefine reconstructive surgery, enabling the generation of functional, vascularized tissue constructs with reduced donor-site morbidity.

Despite remaining challenges, including procedural complexity and the need for standardized clinical protocols, the approaches discussed in this review represent a promising pathway toward the routine clinical application of bone tissue engineering strategies.

## Figures and Tables

**Table 1:** Biological characteristics, advantages, limitations, and ideal clinical indications of vascularized flap-based and axial vascularization strategies in bone tissue engineering.

Strategy	Biological Characteristics	Advantages	Limitations	Ideal Indication
Periosteal flap	Osteogenic and highly vascularized	High osteogenicity	Limited tissue volume	Segmental bone defects
Muscle flap	Highly vascularized soft tissue	High vascularity	Low intrinsic osteogenesis	Prefabricated constructs
Omentum flap	Angiogenic and adaptable tissue	Adaptability	Requires abdominal surgery	Irregular or complex defects
AV loop	Intrinsic axial vascularization	Controlled vascular network	Microsurgical complexity	Large or prefabricated constructs

**Table 2:** Representative preclinical, translational, and clinical studies investigating vascularized flaps, axial vascularization, and in vivo bioreactor strategies in bone tissue engineering.

Author	Model	Approach	Scaffold	Biological Strategy	Vascularization Type	Key Findings
**Biological flaps**						
Huang et al., 2017 [12]	Rabbit (in vivo)	In vivo bioreactor (periosteal flap)	DBM	Periosteum-derived osteogenic cells	Intrinsic (pedicled flap)	Increased bone mass, vascular density, and biomechanical strength compared to muscle pouch
Nau et al., 2017 [13]	Rat (critical femoral defect)	Axial vascularization + tissue engineering	β-TCP	MSCs + EPCs	Axial (periosteal flap)	Enhanced BMD, angiogenesis, and mechanical stability; vascular supply is essential
Naujokat et al., 2022 [25]	Pig (omentum model)	Ectopic in vivo bioreactor	Ceramic scaffold + BMP	Growth factors + omental environment	Intrinsic (omentum-based vascularization)	Successful bone formation; minimally invasive robotic technique feasible
Sparks et al., 2022 [49]	Sheep (preclinical proof of concept)	Scaffold + corticoperiosteal flap	mPCL-TCP	CPF (osteogenic progenitors + growth factors)	Axial	Effective regeneration even in large defects
Sparks et al., 2023 [24]	Sheep + clinical case	RMAV (advanced axial vascularization)	mPCL-TCP	Corticoperiosteal flap	Intrinsic axial	Bone regeneration comparable to autograft; successful clinical translation
Watson et al., 2020 [47]	Sheep (large animal)	In vivo bioreactor adjacent to rib periosteum	Autograft/Bio-Oss	Periosteal induction ± infection modulation	Intrinsic (periosteum driven)	Successful bone formation; infection influences regeneration
Warnke et al., 2004 [16]	Human (clinical case)	In vivo bioreactor (latissimus dorsi muscle flap prefabrication)	Titanium mesh + bone mineral blocks	rhBMP-7 + bone marrow	Intrinsic (muscle flap)	First successful prefabrication and transplantation of a vascularized mandibular bone graft in humans
Heliotis et al., 2006 [50]	Human (clinical case)	In vivo bioreactor (prefabricated pedicled bone flap)	Hydroxyapatite scaffold + OP-1 (BMP-7)	Osteoinductive stimulation within vascularized environment	Intrinsic (pedicled flap)	Successful transformation of scaffold into a vascularized bone flap demonstrating clinical feasibility of in vivo bone tissue engineering
Kokemüller et al., 2010 [51]	Sheep (preclinical) + clinical application	In vivo bioreactor (muscle-based prefabrication)	β-TCP scaffold + autologous bone marrow	Osteogenic cell loading + axial vascular bundle	Intrinsic axial (vascular bundle)	Axial perfusion significantly enhanced angiogenesis, bone formation, and scaffold integration; successful translation toward mandibular reconstruction
**AV loop and axial vascularization**						
Kneser et al., 2006 [18]	Rat (in vivo)	AV loop + isolation chamber	Porous cancellous bone matrix (PBCB)	Intrinsic vascular induction	Intrinsic axial (AV loop)	Effective axial prevascularization of bone scaffold with organized microvascular network supporting subsequent bone tissue engineering
Arkudas et al., 2007 [9]	Rat	Scaffold + vascular pedicle	Fibrin glue-coated PCL scaffold	BMSCs	Intrinsic axial (vascular pedicle-AV loop-related)	Enhanced vascularization and bone formation within scaffold-based constructs
Arkudas et al., 2007 [19]	Rat	Axial prevascularization (AV loop)	Porous scaffold	Autologous osteoblasts	Intrinsic axial (AV loop)	Improved survival and differentiation of transplanted osteoblasts
Paré et al., 2022 [20]	Sheep	AV loop + mandibular defect	Biphasic CaP scaffold	Bone marrow loading	Intrinsic axial	Full osseointegration and lamellar bone formation
Han & Dai, 2013 [52]	Rabbit	Intrinsic vascularized in vivo bioreactor (vessel bundle)	β-TCP	BMP-2-transduced MSCs	Intrinsic axial (AV bundle)	Bone formation observed only in presence of osteoinductive stimulus (BMP-2)
Horch et al., 2014 [53]	Human (clinical cases)	In situ bone tissue engineering with AV loop	Cancellous bone + fibrin glue/β-TCP-HA + fibrin glue	Autologous bone graft or bone marrow aspirate	Intrinsic axial vascularization (AV loop)	Long-term healing of large radius and tibial defects with patent AV loops and stable bone regeneration at 36–72 months
Weigand et al., 2015 [54]	Sheep (large animal)	AV loop + perforated/isolation chamber	Nanostructured bone substitute	Scaffold remodeling with intrinsic/extrinsic vascular ingrowth	Intrinsic axial vascularization ± extrinsic vascularization	Combined intrinsic/extrinsic vascularization accelerated scaffold vascularization, tissue ingrowth, remodeling, and bone formation in clinically relevant constructs
**Hybrid Strategies**						
Gonzalez Matheus et al., 2022 [55]	Clinical (feasibility trial)	Hybrid RMAV approach	mPCL-TCP (3D-printed scaffold)	Corticoperiosteal flap (osteogenic and vascular source)	Intrinsic axial	Proposed regenerative matching axial vascularization strategy for large calvarial defects; ongoing clinical translation

Abbreviations: AV, arteriovenous; BMD, bone mineral density; BMP, bone morphogenetic protein; BMSCs, bone marrow stromal cells; CaP, calcium phosphate; CPF, corticoperiosteal flap; DBM, decellularized bone matrix; EPCs, endothelial progenitor cells; MSCs, mesenchymal stem cells; mPCL-TCP, medical-grade polycaprolactone–tricalcium phosphate; OP-1, osteogenic protein-1; PBCB, porous cancellous bone matrix; PCL, polycaprolactone; RMAV, regenerative matching axial vascularization; rhBMP-7, recombinant human bone morphogenetic protein-7; β-TCP, beta-tricalcium phosphate.

**Table 3:** Proposed decision-making framework for selecting vascularization strategies in scaffold-based bone reconstruction.

Reconstructive Setting	Preferred Strategy	Biological Rationale	Potential Application
Small bone defects with preserved vascularity	Autologous bone grafting or conventional scaffold-based reconstruction	Native vascular supply is generally sufficient to support graft integration and bone healing	Standard reconstructive approach for limited defects
Large defects with adequate soft tissue coverage	Scaffold-based reconstruction with biological augmentation	Osteoconductive scaffolds may support regeneration when surrounding vascularity is preserved	Suitable for selected large but biologically favorable defects
Large defects associated with poor vascularity or soft tissue loss	Vascularized flap, preferably periosteal, combined with scaffold-based reconstruction	Provides immediate perfusion together with osteogenic and angiogenic biological support	Particularly useful in compromised recipient beds
Complex or critical-size defects	Hybrid strategies integrating vascularized flaps, scaffolds, and osteoinductive stimuli	Combines structural stability, vascularization, and biological stimulation to enhance regeneration	Advanced reconstructive option for challenging defects
Extensive defects or prefabricated constructs	AV loop-based axial vascularization combined with scaffold-based engineering	Establishes intrinsic and homogeneous vascular perfusion throughout the construct	Promising strategy for large-scale tissue-engineered reconstruction
Irregular craniofacial or anatomically complex defects	Omental flap-based or adaptable vascularized bioreactor strategies	Highly vascularized and conformable tissues adapt to complex three-dimensional geometries	Potentially useful in personalized craniofacial reconstruction

## Data Availability

No new data were created or analyzed in this study. Data sharing is not applicable to this article.

## Abbreviations

The following abbreviations are used in this manuscript:

AV	Arteriovenous
BMP	Bone Morphogenetic Protein
BMD	Bone Mineral Density
BMSCs	Bone Marrow Stromal Cells
CaP	Calcium Phosphate
CPF	Corticoperiosteal Flap
DBM	Decellularized Bone Matrix
EPCs	Endothelial Progenitor Cells
MeSH	Medical Subject Headings
MSCs	Mesenchymal Stem Cells
mPCL-TCP	Medical-grade Polycaprolactone–Tricalcium Phosphate
PBCB	Porous Cancellous Bone Matrix
PCL	Polycaprolactone
RMAV	Regenerative Matching Axial Vascularization
β-TCP	Beta-Tricalcium Phosphate
